# Characterization of a potential ABC-type bacteriocin exporter protein from *Treponema denticola*

**DOI:** 10.1186/s12903-016-0243-7

**Published:** 2016-07-16

**Authors:** Kimiko Tanaka-Kumazawa, Yuichiro Kikuchi, Yumiko Sano-Kokubun, Seikou Shintani, Masashi Yakushiji, Howard K. Kuramitsu, Kazuyuki Ishihara

**Affiliations:** Department of Pediatric Dentistry, Tokyo Dental College, 2-9-18 Misaki-cho, Chiyoda-ku, Tokyo, 101-0061 Japan; Department of Microbiology, Tokyo Dental College, 2-9-18 Misaki-cho, Chiyoda-ku, Tokyo, 101-0061 Japan; Oral Health Science Center, Tokyo Dental College, 2-9-18 Misaki-cho, Chiyoda-ku, Tokyo, 101-0061 Japan; Department of Oral Biology, State University of New York, Buffalo, NY USA

**Keywords:** ABC transporter, Bacteriocin, Antimicrobial agent susceptibility, *Treponema denticola*

## Abstract

**Background:**

*Treponema denticola* is strongly associated with the development of periodontal disease. Both synergistic and antagonistic effects are observed among bacterial species in the process of biofilm formation. Bacteriocin-related genes have not yet been fully characterized in periodontopathic bacteria. The aim of this study was to detect and characterize bacteriocin-associated proteins in *T. denticola.*

**Methods:**

The whole genome sequence of *T. denticola* ATCC 35405 was screened with a *Streptococcus mutans* bacteriocin immunity protein (ImmA/Bip) sequence. The prevalence of homologous genes in *T. denticola* strains was then investigated by Southern blotting. Expression of the genes was evaluated by qRT-PCR.

**Results:**

In the genome sequence of *T. denticola,* an amino acid sequence coded by the open reading frame TDE_0719 showed 26 % identity with the *S. mutans* ImmA*.* Furthermore, two protein sequences encoded by TDE_0425 and TDE_2431 in *T. denticola* ATCC 35405 showed ~40 % identity with that coded by TDE_0719. Therefore, TDE_0425, TDE_0719, and TDE_2431 were designated as *tepA1*, *A2*, and *A3*, respectively. Open reading frames showing similarity to the HlyD family of secretion proteins were detected downstream of *tepA1*, *A2*, and *A3.* They were designated as *tepB1*, *B2*, and B3, respectively. A gene harboring a bacteriocin-like signal sequence was detected upstream of *tepA1.* The prevalence of *tepA1* and *A2* differed among *Treponema* species. Susceptibility to chloramphenicol and ofloxacin was slightly decreased in a *tepA2* mutant while that to kanamycin was increased. Expression of *tepA3-B3* was increased in the *tepA2* mutant.

**Conclusion:**

These results indicate that *T. denticola* ATCC 35405 has three potential bacteriocin export proteins and that the presence of these genes differs among the *Treponema* strains. TepA3-B3 of the corresponding proteins may be involved in resistance to chloramphenicol.

## Background

*Treponema denticola* is a spiral-shaped motile rod that is frequently isolated from the periodontal pockets of subjects with chronic periodontitis [1, 2] and that possesses several potential virulence factors [3]. It is often co-isolated with *Porphyromnas gingivalis* and *Tannerella forsythia* from the dental plaque biofilms of chronic periodontitis patients [4] and is involved in the development of periodontitis [5, 6].

Interactions among bacterial species that reside in dental plaque biofilms influence the composition of the biofilms [7–9]. Microorganisms in biofilms develop their niche by symbiosis with other microorganisms and suppression of competitors by secreting antagonistic factors [10, 11]. To suppress the growth of a competitor, various microorganisms produce antimicrobials such as bacteriocins and H_2_O_2_ [12]. These antimicrobial factors play an important role in the survival strategy of the microorganisms in the biofilms. Similarly, in dental plaque biofilms, many species of microorganisms have been reported to produce bacteriocins or bacteriocin-like substances [13–18]. In the bacteriocin-producing bacteria, genes encoding a bacteriocin, a bacteriocin ABC transporter, and bacteriocin immunity proteins are involved in bacteriocin production [19]. Bacteriocin immunity proteins protect the microbes against the effects of their own bacteriocins [20]. Bacteriocin ABC transporters have two functional domains: a peptidase C39 domain, which is involved in the processing of bacteriocin precursors at the double glycine, and an ABC transporter domain, which is involved in the export of the bacteriocins [21]. A synergistic effect between *T. denticola* and *P. gingivalis* has been reported [22], while growth of *T. denticola* was inhibited by plaque-associated *Streptococcus mutans* [23]. The antagonistic effects produced by bacteriocins have been genetically characterized in oral streptococci [24–26], while those in periodontopathic bacteria remain to be established. Suppressing the growth of and avoiding inhibition by competitors would benefit *T. denticola* in the colonization of the subgingival plaque and the development of periodontopathic biofilms.

In the present study, we intended to characterize bacteriocin-associated proteins from *T. denticola.* By screening of *T. denticola* genomic DNA using *S. mutans* bacteriocin immunity protein, ABC-type bacteriocin exporter-like proteins were detected and the function of the exporter was investigated.

## Methods

### Bacterial strains and culture conditions

The strains of *T. denticola* used in this study are listed in Table 1. The strains were maintained in TYGVS medium [27] at 37 °C under anaerobic conditions (10 % CO_2_, 10 % H_2_, and 80 % N_2_) in an anaerobic chamber (Hirasawa, Tokyo, Japan). For the mutant strain, TYGVS containing 40 μg/ml erythromycin was used.

**Table 1:** List of the strains used in this study

Bacterial strain	Relevant characteristics	Source or reference
*T. denticola* ATCC 33520	Em^s^	[41]
*T. denticola* ATCC 33521	Em^s^	[41]
*T. denticola* ATCC 35404	Em^s^	[41]
*T. denticola* ATCC 35405	Em^s^	[41]
*T. denticola* GM1	Em^s^	[42]
*T. denticola* KT-3	*tepA2*:: Em^r^	This study

### Sequence homology-based screening

The whole genome sequence of *T. denticola* ATCC 35405 in the Los Alamos oral pathogen database (http://www.oralgen.org) was screened for homologous sequences with the *S. mutans* bacteriocin immunity protein (ImmA/Bip) sequence [28] using the protein blast program. The obtained homologous sequences were further compared against the database of National Center for Biotechnology Information (NCBI, http://blast.ncbi.nlm.nih.gov/Blast.cgi). The DNA sequences coding for the homologous proteins in *T. denticola* were designated as *tepA1, A2, and A3* as described in the Results section, and they were characterized with Genetyx-MAC v. 17.0.6 (Genetyx Corporation, Tokyo, Japan).

### Southern blotting

The prevalence of DNA sequences homologous to *tepA1*, *A2*, and *A3* in *T. denticola* species was detected by Southern blotting. *T. denticola* was grown for 3 days in TYGVS medium and genomic DNA was isolated with the Gentra Puregene Cell Kit (Qiagen, Tokyo, Japan). Southern blot analysis was performed as described previously [29]. Digoxigenin-labeled primer was synthesized on a GeneAmp 9700 thermal cycler (Life Technologies, Carlsbad, CA) with the PCR DIG Probe Synthesis Kit (Roche Diagnostics, Tokyo, Japan) according to the manufacturer’s instructions using the 3 primer pairs I-1F and I-1R, I-2F and I-2R, and I-3F and I-3R, for *tepA1*, *tepA2*, and *tepA3*, respectively (Table 2). Hybridized bands were detected with the DIG Nucleic Acid Detection Kit (Roche Diagnostics).

**Table 2:** List of gene-specific primers used in this study

Primers and probes	Sequence
I-1 F	5′-AAATTTGCAAAGGCCTACCGTGAGCTT-3′
I-1R	5′-TTTCGACAAAAGAGTGTACTCCCGTTTCC-3′
I-2 F	5′-CGGGCGGTATACTCATGCTGATTGCC-3′
I-2R	5′-TTTGCCTGAACCGGCTCCTAC-3′
I-3 F	5′-CGGAGAACTTGTTGCAAGGATGAACGATAC-3′
I-3R	5′-TCCCGAAAAACAAGAGAATGTCCTGAGGAAC-3′
718D2	5′-GGTTTGCTCTTGCAATTCCCATATTTA-3′
719U	5′-**TGTTGCAAATACCGATGAG**CAAAATAATATGAGAACGCACCGCAGAA-3′
EMD2	5′-GCTCATCGGTATTTGCAACATCATAG-3′
EMU2	5′-CTACATTCCCTTTAGTAACGTGTAACTTTC-3′
719D	5′-**CGTTACTAAAGGGAATGTAG**CTATTTTGACGGGTTGGAGTTCCAACA-3′
720U	5′-TACGGTACCTGAATAAGCAGCCTTACC-3′
tepA1F	5′-TGCCGTGCAAATGACTCTCT-3′
tepA1R	5′-TTTTAAAACTGCCTACCCAATAAACGC-3′
tepA1P^a^	5′-CACAGCTTGGAACTTT-3′
tepB1F	5′-TGAAAAAATTATGGCTTGAAGCACTTGA-3′
tepB1R	5′-TGCCATATCTGCCTTGTATTTTAACTCT-3′
tepB1P^a^	5′-CCTGCAACAGCAATTC-3′
tepA3F	5′-CACTCCTGTATTGTTGAAAGTCTTAACG-3′
tepA3R	5′-CACTTACGATTTTAAACTCGGCTCTT-3′
tepA3P^a^	5′-TTGGGTGCCGAATCTA-3′
tepB3F	5′-AGAAAGTTTAAAACTTTTTACAGTCTATGCTCCT-3′
tepB3R	5′-CATTATCCCCGCAGTTAAGAGATGA-3′
tepB3P^a^	5′-TTCCTGCACTTCTCCC-3′
tetRF	5′-CGCAACGCCGGTTCTTAAAA-3′
tetRR	5′-CCTTCGAACAACAGACAATCAGTTT-3′
tetRP^a^	5′-TCGCATCCCAATTATC-3′
16SF	5′-GCCGATGATTGACGCTGATATAC-3′
16SR	5′-CGGACTACCAGGGTATCTAATCCT-3′
16SP	5′-CTCCCCGCACCTTC-3′

Boldface sequences overlap with the 5′ or 3′ end of *ermF-ermAM*, ^a^Taqman probe

### Construction of a *tepA2* mutant

As TepA2 showed similarity to ImmA, a *tepA2-*deficient mutant of *T. denticola* ATCC 35405 was constructed by allelic exchange mutation to investigate the role of *tepA2*. Briefly, two fragments flanking the *tepA2* gene were amplified with primer pairs 718D/719U and 719D/720U (listed in Table 2), respectively. The *ermF-ermAM* cassette was amplified with the primers EMD2 and EMU2 and the fragment was inserted between the upstream and downstream fragments using the PCR-based overlap-extension method [30]. The constructed fragments were introduced by electroporation and transformants were isolated on TYGVS agar plates containing 40 μg/ml erythromycin as described previously [29]. Inactivation of the gene in mutant KT-3 was confirmed by Southern blot and PCR analyses.

### Antibiotic susceptibility testing

The effect of *tepA2* inactivation on the susceptibility of *T. denticola* to antibiotics was investigated. Chloramphenicol, ofloxacin, and kanamycin, to which *T. denticola* showed low susceptibility in our preliminary results, were selected. *T. denticola* ATCC 35405 and KT-3 were cultured as described above for 4 days. The cells were adjusted to an optical density at 660 nm (OD_660_) of 0.1 with TYGVS medium using a spectrophotometer (UV-2550, Shimadzu, Kyoto, Japan), and 100 μl of the cell suspension was added to TYGVS containing 0.5–1 μg/ml of chloramphenicol, 16–64 μg/ml of kanamycin, or 8–32 μg/ml of ofloxacin. After incubation for 7 days under anaerobic conditions, cell growth was measured at OD_660_ with the spectrophotometer.

### DNA microarray analysis

*T. denticola* ATCC 35405 and KT-3 were cultured as described above for 2 days. To investigate the relation between inactivation of *tepA*2 and increase of chloramphenicol resistance, exponentially growing cells (OD_660_ ~ 0.2) were incubated with chloramphenicol (1 μg/ml) for 4 h. The cells were harvested immediately after chloramphenicol treatment and total RNA was extracted using Trizol (Life Technologies). DNase treatment was carried out using a TURBO DNA-free kit Life Technologies). cDNA was synthesized using a SuperScript Double-Stranded cDNA Synthesis kit (Invitrogen). DNA microarray gene expression analysis was carried out using Roche NimbleGen custom arrays (2006-07-27_TI243275_60mer; Roche, Indianapolis, IN) according to the standard NimbleGen procedure (NimbleGen arrays user’s guide: gene expression analysis, v6.0). Briefly, cDNA (0.5–1 μg) was labeled using a NimbleGen one-color labeling kit, in which Cy3 was randomly incorporated into the newly synthesized DNA by the Klenow fragment. Labeled cDNA (3 μg) derived from each RNA sample was hybridized with each array for 16 to 18 h. The slides were washed, spun dry, and scanned with an Agilent Microarray Scanner with a resolution of 5 μm. Normalization was carried out with the NimbleScan 2.6.0.0 built-in normalization function. Target genes were confirmed by real-time PCR analysis using primers listed in Table 2.

### Quantitative reverse transcription (qRT) PCR expression analysis of *tepA1-B1*, *tepA3-B3*, and TDE_0820

To investigate the relationships among *tepA1, A2* and *A3*, expression of *tepA1* and A*3, tepB1* and *B3,* and TDE_0820 in the wild-type strain and KT-3 were evaluated. *T. denticola* ATCC 35405 and KT-3 were cultured as described above. Cells at mid-log phase (OD_660_ of 0.4–0.6) were harvested and total RNA was extracted using Trizol (Life Technologies). DNase treatment was carried out using a TURBO DNA-free kit (Life Technologies) and cDNA was synthesized using ReverTra Ace (Toyobo, Osaka, Japan). Gene expression was measured with real-time PCR using primers and the Taqman probe (Life Technologies) listed in Table 2 on a 7500 Real-Time PCR System (Life Technologies). Expression of each gene was normalized to the level of 16S rRNA as an internal control and was expressed as a fold modulation relative to the wild-type strain grown without chloramphenicol.

### Statistical analysis

Comparisons of gene expression and susceptibility to antibiotics were carried out using Student’s *t*-test. One-way ANOVA followed by Tukey’s multiple comparison test was used for comparisons of gene expression among the two strains grown with or without chloramphenicol. All tests were carried out using Prism v. 5f (GraphPad software, San Diego, CA). The level of significance for all statistical tests was set at *P* < 0.05.

## Results

### Screening of homologous sequences for bacterial immunity proteins in *T. denticola*

**Fig. 1:**
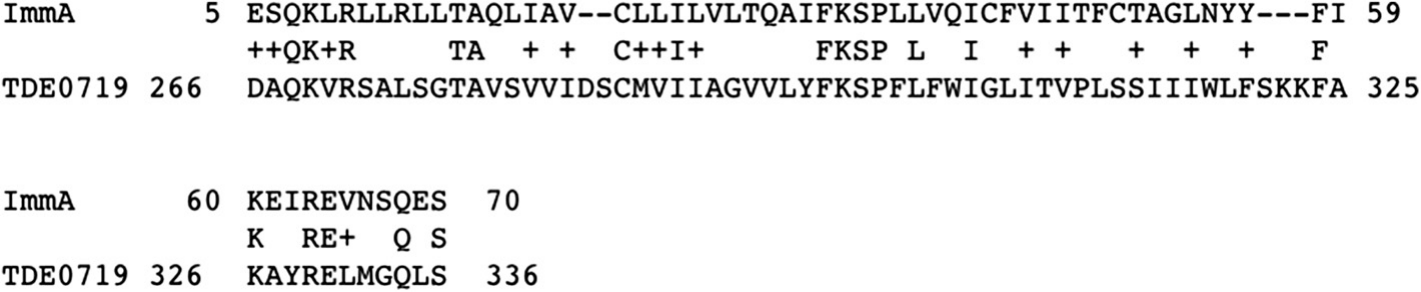
Homology between the *S. mutans* bacteriocin immunity protein ImmA and the deduced amino acid sequence of TDE_0719 in *T. denticola* ATCC35405

A search against the *T. denticola* ATCC 35405 whole genome sequence for sequences homologous to *S. mutans* ImmA revealed an amino acid sequence that possesses 26 % identity with ImmA between residues 5 and 70 (Fig. 1). The sequence is coded by a 2154-bp open reading frame, TDE_0719, and consists of 717 amino acids. The calculated molecular mass of the deduced amino acid sequence is 80279.25, and the estimated pI is 8.76. The TDE_0719 sequence was compared to the NCBI nucleotide sequence database. The DNA sequence from bp 1408 to 2059, which codes for amino acid residues 469 to 686, shows 60–70 % identity with ABC transporters of *T. denticola* ATCC 35405, *Clostridium botulinum*, and *Spirochaeta caldaria*. This region encodes part of a potential ATP-binding site. The amino acid sequence shows 45 % identity with the bacteriocin ABC transporter of *Spirochaeta africana* DSM 8902 in a 713-amino-acid overlap, 46 % identity with that of *Clostridium lentocellum* DSM 5427 in a 713-amino-acid overlap, and 47 % identity with that of *Clostridium clariflavum* DSM 19732 in a 710-amino-acid overlap. In addition to these sequences, amino acid sequences deduced from TDE_0425 and TDE2431 in *T. denticola* ATCC 35405, which were not detected in the first screening using *S. mutans* ImmA, also show homology with that coded by TDE_0719 (43 % in a 715-amino-acid overlap and 40 % in a 710-amino-acid overlap, respectively).

**Fig. 2:**
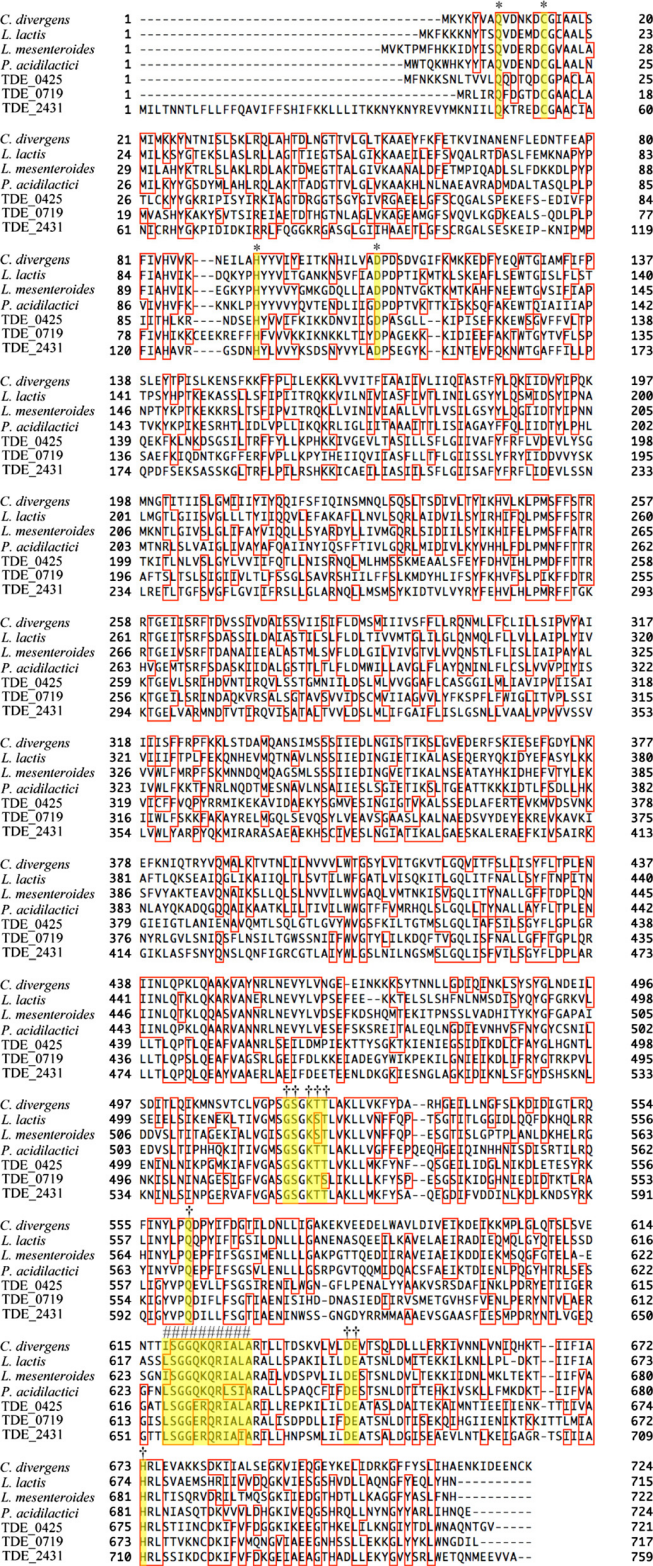
Multiple sequence alignments of bacteriocin ABC transporters. Cysteine and histidine, which are part of the putative active site of the peptidase family C39B, as well as glutamine, which contributes to the oxyanion pore in other cysteine protease families, are marked with * and indicated in yellow. The ATP-binding site and ABC transporter signature motifs are indicated in yellow and marked with † and #, respectively. The alignment was carried out using the program Genetyx-Mac 16.0.9. *C. divergens*: ATP-dependent transporter of *Carnobacterium divergens*, *L. lactis*: Lactococcin-A transport/processing ATP-binding protein LcnC of *Lactococcus lactis* subsp. *lactis, L. mesenteroides*: Mesentericin-Y105 transport/processing ATP-binding protein MesD of *Leuconostoc mesenteroides, P. acidilactici*: Pediocin PA-1 transport/processing ATP-binding protein PedD of *Pediococcus acidilactici,* TDE_0425: *tepA1*, TDE_0719: *tepA2*, TDE_2431: *tepA3*

A search of the NCBI Conserved Domain Database (http://www.ncbi.nlm.nih.gov/Structure/cdd/cdd.shtml) revealed that amino acid residues 1–140 of TDE_0719 display similarity to the peptidase C39B domain, residues 160–420 to the ABC membrane superfamily, and residues 480–717 to the P-loop NTPase superfamily. Domains of the ABC membrane superfamily and the P-loop NTPase superfamily are common features of bacterial transporters [31]. Multiple comparisons with other bacteriocin ABC transporters are depicted in Fig. 2. The Q, C, and H residues form the putative active site of a subfamily of the peptidase family C39, which mostly consists of bacteriocin-processing endopeptidases from bacteria [32], and are conserved in the corresponding regions of TDE_0425, TDE_0719, and TDE2431. The ABC transporter signature motif LSGGQRQRIA and GS-KTT, Q, DE, and H, which are observed at ATP-binding sites [33], were highly conserved in the corresponding regions of the *T. denticola* sequences. We designated the three open reading frames TDE_0425, TDE_0719, and TDE2431 as *Treponema* exporter proteins A1, A2, and A3 (*tepA1*, *tepA2*, and *tepA3* genes), respectively.

TDE_0720, which is located immediately downstream of TDE_0719, displayed similarity to the multidrug resistance efflux pump of *Spirochaeta africana* DSM 8902 (22 % identity in a 369-amino-acid overlap) and the HlyD family secretion protein of *Desulfosporosinus* sp. OT (27 % identity in a 459 amino-acid overlap). The region of TDE_0720 spanning residues 240 to 350 had similarity to a domain of a HlyD family secretion protein (pfam13437) that is reported to be part of an accessory protein for ABC exporters of gram-negative bacteria for translocating proteins across the outer membrane [31]. Downstream of *tepA1* and *tepA3*, TDE_0426 and TDE2430 were detected, and both also showed similarity to sequences coding HlyD family secretion proteins. The presence of the C39B peptidase domain, a conserved ATP-binding motif, a membrane-spanning domain, and a nearby accessory protein facilitating export is consistent with the properties of a bacterial ABC exporter. Therefore, we designated TDE_0426, TDE_0720, and TDE2430 as *Treponema* exporter proteins B1, B2, and B3 (*tepB1*, *tepB2*, and *tepB3*), respectively*.*

Bacteriocin transporters are reported to cleave the double-glycine leader peptides from the precursors of bacteriocins for their secretion [31]. A search of the flanking regions of *tepA1*, *A2*, *and A3* in the genome sequence of *T. denticola* ATCC 35405 revealed that proteins coded by three open reading frames (TDE_0416, TDE_0422, and TDE_0423) upstream of *tepA1* have double-glycine bacteriocin-type signal domains. However, no double glycine-containing protein-coding sequence exists near *tepA2* and *A3.* Of the three proteins, those coded by TDE_0422 and TDE_0424 showed high overall similarity (92 %). The protein coded by TDE_0416 showed high sequence identity with TDE_0422 and TDE_0424 (98 % and 84 %, respectively); however, the sequence was truncated at residue 167. TDE_0422 and TDE_0424 showed only weak similarity to penicillin-binding proteins of *Bacillus cereus* VD148 (37 % identity in a 89-amino-acid overlap).

### Prevalence of *tepA1*, *A2*, and *A3* in *T. denticola* strains

**Fig. 3:**
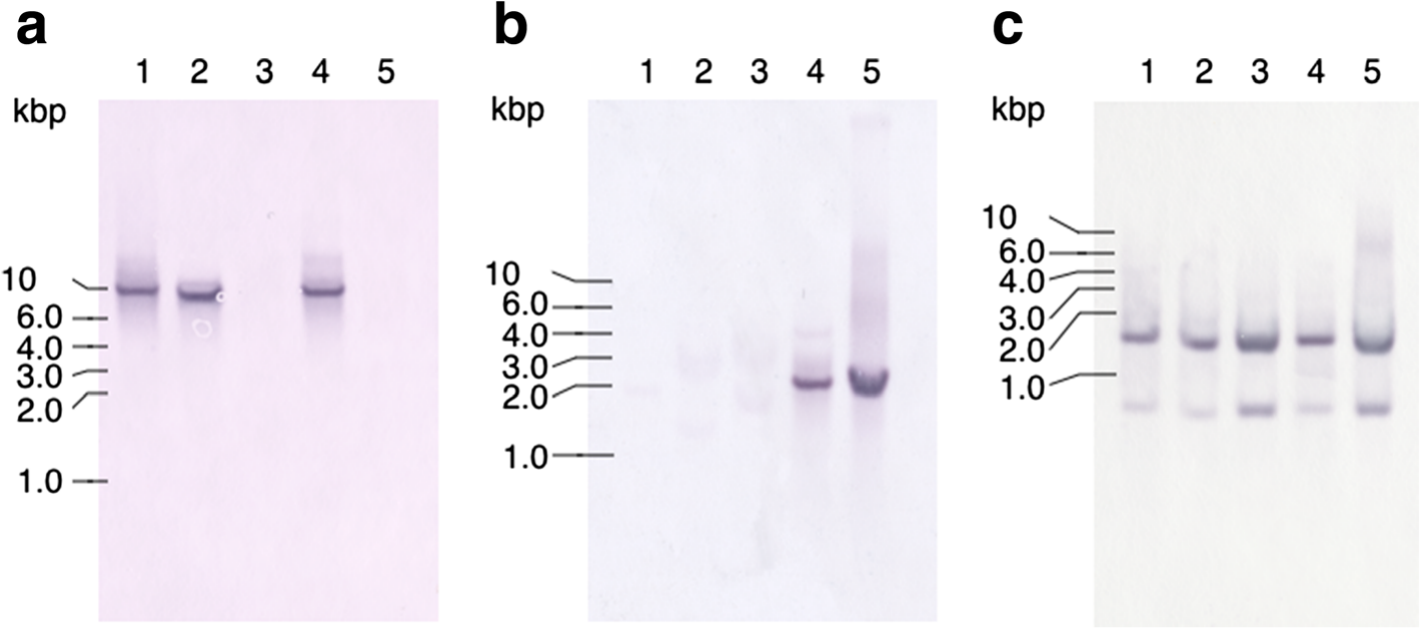
Southern blot analysis of *tepA1* (**a**), *tepA2* (**b**)*,* and *tepA3* (**c**). Genomic DNA from *T. denticola* strains was digested with *Hin*dIII. 1: genomic DNA from ATCC 33520, 2: genomic DNA from ATCC 33521, genomic DNA from ATCC 35404, 4: genomic DNA from ATCC 35405, 5: genomic DNA from GM1

We examined the presence of the three *tepA1*, *A2*, and *A3* sequences in *T. denticola* strains ATCC 33520, ATCC 33521, ATCC 35404, ATCC 35405, and GM1 by Southern blotting (Fig. 3). As a single band of approximately 9 kbp was detected in ATCC33520, ATCC33521, and ATCC 35405 with the *tepA1* probe; a band at approximately 2 kb was detected from ATCC 35405 and GM1 with the *tepA2* probe; and all strains exhibited two bands at approximately 1.5 kb and 0.8 kb with the *tepA3* probe.

### Antimicrobial sensitivity of the *tepA2-*deficient mutant

**Fig. 4:**
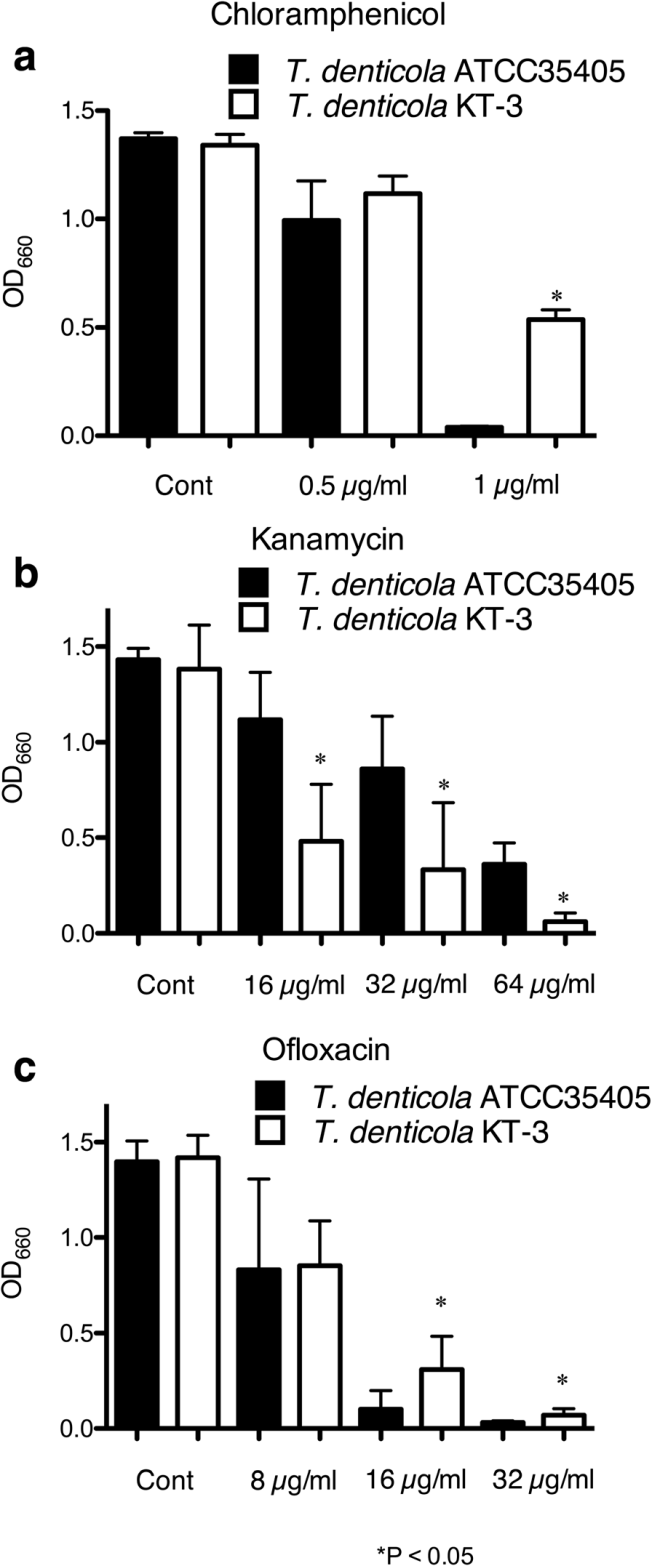
Effect of inactivation of *tepA2* on the growth of *T. denticola* in medium containing chloramphenicol (**a**), kanamycin (**b**), or ofloxacin (**c**) in the wild-type and *tepA2-*deficient KT-3 strains. *T. denticola* was adjusted to OD_660_ = 0.1 and inoculated into TYGVS medium containing antibiotics. Growth of *T. denticola* was evaluated by measuring the OD_660_. The experiments were performed twice in quadruplicate. Data are presented as the mean ± SD (*n* = 8). *P < 0.05 vs. ATCC 35405 under the same concentration of antibiotics

Among the three bacteriocin transporter-like sequences, we focused on TepA2 because it showed the highest similarity to ImmA. Bacteriocin-like sequences were not detected around *tepA2* and *B2*. Therefore, it is possible that the protein transports other molecules in addition to a bacteriocin. To characterize the function of *tepA2* and the functional interaction among the three exporter genes, a *tepA*2-deficient mutant of strain ATCC 35405 was constructed and designated KT-3. Inactivation of *tepA2* did not affect the growth of *T. denticola* (data not shown). Various ABC export systems transport non-protein molecules such as lipophilic drugs, antibiotics, and polysaccharides [31]. To investigate the possible involvement of *tepA*2 in antibiotic export, the susceptibility of strain 35405 against chloramphenicol, kanamycin, and ofloxacin was evaluated. In the presence of 1 μg/ml chloramphenicol, the growth of the wild type was completely inhibited while *T. denticola* KT-3 showed 40.0 % growth relative to the control (Fig. 4). In the presence of 16–64 μg/ml of kanamycin, the growth of *T. denticola* KT-3 was significantly lower than that of the wild-type strain. In the presence of 16–32 μg/ml of ofloxacin, the growth rate of *T. denticola* KT-3 was higher than that of the wild-type. These results indicated that inactivation of *tepA2* might influence the susceptibility of *T. denticola* to these antimicrobials.

### DNA microarray analysis of the *tepA2-*deficient mutant

In the *tepA2-*deficient mutant, susceptibility against kanamycin decreased while that to chloramphenicol and ofloxacin increased. To investigate the mechanisms of the increase in susceptibility against chloramphenicol, the gene expression profiles of *T. denticola* ATCC 35405 and KT-3 during exposure to chloramphenicol were compared using microarrays. Significantly differentially expressed genes in KT-3 relative to the wild-type strain are shown in Tables 3 and 4. Expression of the GNAT family acetyltransferase, glucosamine-6-phosphate deaminase, precorrin-4 C11-methyltransferase, several ABC transporters, and potential transcriptional regulators including TDE_0820 (transcriptional regulator, TetR family) was increased, while that of methyltransferase domain protein, several ABC transporters, and GGDEF domain proteins, as well as pyrrolidone-carboxylate peptides were decreased. Increases in expression of *tepA1* and *tepA3* were detected in the mutant; however, they were weak when compared to those of the listed genes.

**Table 3:** Genes with increased expression in the *tepA2*-deficient mutant in the presence of chloramphenicol

Gene	Gene expression fold change (KT-3 versus wild type)
TDE_0499 hypothetical protein	50.5
TDE_2748 acetyltransferase, GNAT family	30.0
TDE_0337 glucosamine-6-phosphate deaminase	19.5
TDE_2214 hypothetical protein	17.9
TDE_0561 hypothetical protein	16.0
TDE_0614 precorrin-4 C11-methyltransferase	16.0
TDE_0506 DNA-damage-inducible protein J, putative	15.3
TDE_1848 hypothetical protein	15.2
TDE_0307 hypothetical protein	14.3
TDE_2378 ABC transporter, ATP-binding protein, putative	14.2
TDE_0259 transcriptional regulator, MarR family	12.7
TDE_1599 ABC transporter, ATP-binding/permease protein	12.5
TDE_0551 hypothetical protein	11.4
TDE_0820 transcriptional regulator, TetR family	11.3
TDE_1517 hypothetical protein	11.1
TDE_1692 hypothetical protein	11.1
TDE_0528 hypothetical protein	11.0
TDE_0475 ABC transporter, ATP-binding protein	11.0
TDE_2519 hypothetical protein	10.5
TDE_0231 DNA polymerase III, beta subunit	10.3
TDE_0382 hypothetical protein	9.8
TDE_2638 hypothetical protein	9.5
TDE_0375 ABC transporter, ATP-binding protein	9.4
TDE_1977 hypothetical protein	9.3
TDE_0748 iron compound ABC transporter, periplasmic iron compound-binding protein, putative	8.8
TDE_0426 bacteriocin ABC transporter, ATP-binding/permease protein, putative	6.1
TDE_2431 bacteriocin ABC transporter, ATP-binding/permease protein, putative	2.8

**Table 4:** Genes with decreased expression in the *tepA2*-deficient mutant in the presence of chloramphenicol

	Gene expression fold change (KT-3 versus wild type)
TDE_0719 bacteriocin ABC transporter, ATP-binding/permease protein, putative	0.0004
TDE_1057 hypothetical protein	0.02
TDE_1181 methyltransferase domain protein	0.03
TDE_2761 hypothetical protein	0.05
TDE_0953 branched-chain amino acid ABC transporter, permease protein	0.05
TDE_1883 hypothetical protein	0.06
TDE_1066 hypothetical protein	0.06
TDE_2582 GGDEF domain protein	0.06
TDE_1058 hypothetical protein	0.06
TDE_0720 bacteriocin ABC transporter, bacteriocin-binding protein, putative	0.07
TDE_0998 hypothetical protein	0.09
TDE_1930 hypothetical protein	0.1
TDE_0625 ABC transporter, ATP-binding protein	0.1
TDE_0175 pyrrolidone-carboxylate peptidase	0.1
TDE_1921 hypothetical protein	0.1
TDE_0485 hypothetical protein	0.1
TDE_0849 hypothetical protein	0.1
TDE_1446 hypothetical protein	0.1
TDE_0894 hypothetical protein	0.1
TDE_2497 hypothetical protein	0.1
TDE_0912 hypothetical protein	0.1
TDE_0243 ABC transporter, ATP-binding protein	0.1
TDE_2785 hypothetical protein	0.1
TDE_1975 hypothetical protein	0.1
TDE_0485 hypothetical protein	0.1

### Expression of *tepA1-B1*, *tepA3-B3*, and TDE_0820 in the *tepA2* mutant

**Fig. 5:**
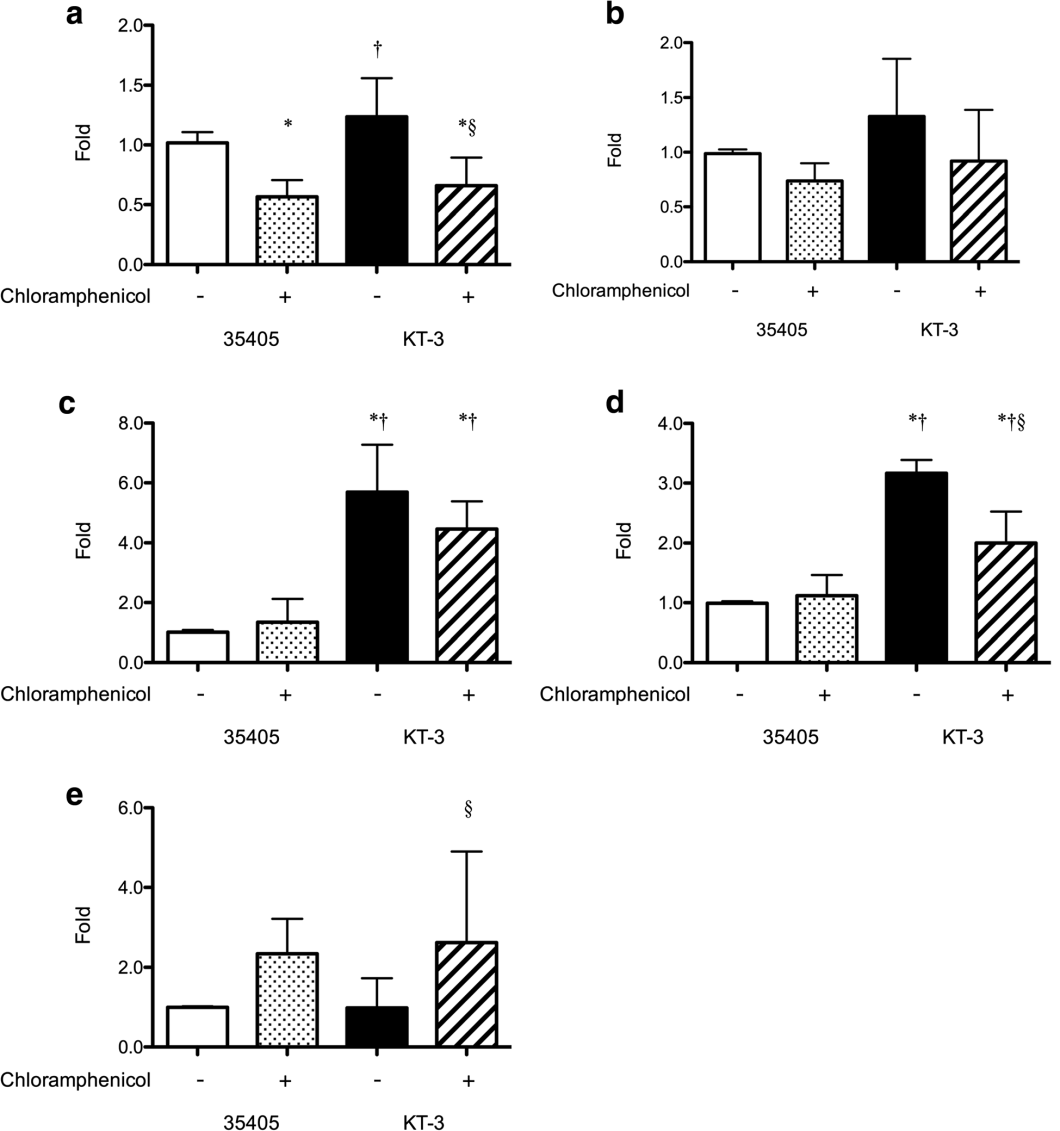
Expression of *tep* and TDE_0820 in the presence or absence of chloramphenicol. Expression of *tepA1* (**a**), *tepB1* (**b**), *tepA3* (**c**)*, tepB3* (**d**), TDE_0820 (**e**) in the presence or absence of chloramphenicol in the wild-type and *tepA2*-deficient mutant KT-3. Expression levels of each gene were normalized using 16S rRNA levels as internal controls and are expressed as a fold modulation relative to the wild-type strain grown without chloramphenicol. Experiments were performed three times in triplicate. Data are presented as the mean ± SD (*n* = 9). *P < 0.05 vs. ATCC 35405 without chloramphenicol, † *P* < 0.05 vs. ATCC 35405 with chloramphenicol, § *P* < 0.05 vs. KT-3 without chloramphenicol

Among the genes that showed increased expression in the presence of chloramphenicol in KT-3 were TDE_0259 (transcriptional regulator, MarR family) and TDE_0820, which code for potential repressor proteins. We selected TDE_0820 for further evaluation by qRT-PCR because the change of expression between the wild type and the mutant was somewhat similar to that of TDE_0259 but its expression level in the wild type and the mutant was approximately 10 times higher than that of TDE_0259. Increases in *tepA1* and *tepA3* expression were detected, although these were lower than those of the other genes in Table 3. To investigate the relationship among the bacteriocin ABC transporter genes, *tepA1-B1*, *tepA3-B3* were also selected for further evaluation by qRT-PCR. The expression of *tepA1* and *tepB1* was not affected by the inactivation of *tepA2* (Fig. 5a and b). The expression of *tepA3* and *tepB3* was significantly increased by inactivation of *tepA2* (Fig. 5c and d), indicating that it is associated with *tepA2-B2* expression. In the presence of chloramphenicol, *tepA1* expression was decreased in both the wild-type strain and KT-3 (Fig. 5a). The expression of *tepA3-B3* did not change with chloramphenicol treatment in the wild-type strain while it decreased in KT-3 (Fig. 5b and c). The expression of TDE_0820 was increased in both the wild-type and KT-3 strains upon chloramphenicol treatment; however, the change was significant only in KT-3 (Fig. 5e). These results indicated that the increased expression of *tepA3-B3* was induced by inactivation of *tepA2* and was independent of exposure to chloramphenicol. Expression of TDE_0820 in KT-3 suggested an association with chloramphenicol sensitivity.

## Discussion

TepA2 shows 42–44 % identity with the bacteriocin exporters of *S. africana* and *C. lentocellum* as well as with two orthologs, TepA1 and TepA3, of *T. denticola* ATCC 35405. The putative active-site residues for bacteriocin peptidases, membrane-spanning domains, and ATP-binding sites were conserved in all of the orthologs. Downstream of the orthologs, sequences for accessory proteins (TepBs), which showed similarity to the functional domain of *hlyd*-like domains, were found. Although bacteriocin production has not been reported in *T. denticola*, putative proteins with double-glycine signal peptides, TDE_0416, TDE_0422, and TDE_0424 were located upstream of *tepA1*. These results tentatively suggest that *tepA-tepB* may code for exporter proteins for these proteins.

The Southern blot analysis results indicated that the number of orthologs of *tepA* differs among *T. denticola* strains. Similarly, diversity among *T. denticola* strains has been reported for Msp (major sheath protein) [34]. However, diversity in the number of orthologs has not yet been documented. Msps are 53–63 kDa and the identity of the amino acid sequences between *T. denticola* strains ATCC 35405 and OKT is 43 % [34]. The amino acid identity among *tepAs* (40 %) was similar to that observed among Msps. Only *tepA3* was detected in all tested strains of *T. denticola.* The genes coding proteins with a bacteriocin-like leader peptide were detected only upstream of *tepA*. It is possible that *tepA* and *tepB*, but not the bacteriocin-like genes, were duplicated during the evolution of *T. denticola* although further analysis is required to clarify the duplication events.

In the *tepA2*-deficient mutant, the expression of *tepA3-B3* increased significantly while that of *tepA1-B1* increased only slightly. In *T. denticola,* regulation of an ABC transporter has been reported only for a thiamine pyrophosphate transporter, which is regulated by a TPP-binding riboswitch [35]. *TepA2-B2* and *tepA3-B3* have similarity to the functional motif for bacteriocin ABC transporters but they are not proximal to bacteriocin-like genes. Only *tepA1-B1* has a bacteriocin-like protein directly upstream. A recent report indicated that peptides secreted through a bacteriocin export system could have signaling functions [36]. It is possible that a reduction in the export of a signaling molecule by *tepA2* may affect the expression of *tepA3-B3*. The expression of *tepA3-B3* increased in the *tepA2* mutant, while that of *tepA1-B1* did not. These results suggest an interaction between regulation of expression of *tepA2-B2* and that of *tepA3-B3*, and that regulation of *tepA1-B1* is independent of *tepA2-B2* and *tepA3-B3.*

Interestingly, resistance to chloramphenicol and ofloxacin was increased while resistance to kanamycin was reduced in the *tep2-*deficient mutant under conditions that induced increased *tepA3* expression. Bacterial efflux pumps, including ABC transporters, are involved in drug resistance in several bacteria [37]. In *T. denticola,* resistance to antimicrobial agents such as human β-defensin 2 and 3, and rifampicin has been reported [38, 39], and an ABC transporter was suggested to be involved in resistance to β-defensin 3. In the microarray analysis, several genes encoding ABC transporters and potential transcriptional regulators including TDE_0820 showed increased expression in the *tepA2* mutant as compared to the wild-type strain. In addition, an acetyltransferase of the GNAT family exhibited significantly increased expression in the *tepA2* mutant. These changes can potentially affect the susceptibility against chloramphenicol. However, the substrate of the enzyme group was reported to be kanamycin [40]; thus, the involvement of the enzyme in chloramphenicol resistance seems unlikely. The expression of *tepA3-B3* in the KT-3 mutant was higher than that in the wild-type strain, although the level was low when compared to that in the mutant without chloramphenicol. It is possible that the changes in the expression of *tepA3-B3* are required for sensitivity to chloramphenicol. Obviously, further analysis is required to define the role of these proteins with putative ABC transporter functions in relation to sensitivity to the three antibiotics tested in this study.

## Conclusions

*T. denticola* ATCC 35405 has three potential bacteriocin export proteins and the presence of these genes differs among the *Treponema* strains. Furthermore, TepA3-B3 of the proteins may be involved in resistance to chloramphenicol. The changes in susceptibility in *T. denticola* may contribute to our knowledge of the use of chemotherapy for chronic periodontitis.

## Abbreviations

ABC transporters, ATP-binding cassette transporters; ImmA, bacteriocin immuniry protein; NCBI, National Center for Biotechnology Information; Tep, *Treponema* exporter protein
